# Acute Hormonal and Inflammatory Responses following Lower and Upper Body Resistance Exercises Performed to Volitional Failure

**DOI:** 10.3390/ijms25137455

**Published:** 2024-07-07

**Authors:** Jakub Chycki, Michał Krzysztofik, Ewa Sadowska-Krępa, Daria Baron-Kaczmarek, Adam Zając, Stanisław Poprzęcki, Miroslav Petr

**Affiliations:** 1 Institute of Sport Sciences, The Jerzy Kukuczka Academy of Physical Education in Katowice, 40-065 Katowice, Poland; m.krzysztofik@awf.katowice.pl (M.K.); e.sadowska-krepa@awf.katowice.pl (E.S.-K.); dariabaron@interia.pl (D.B.-K.); a.zajac@awf.katowice.pl (A.Z.); s.poprzecki@awf.katowice.pl (S.P.); 2 Faculty of Physical Education and Sport, Charles University, 162 52 Prague, Czech Republic; petr@ftvs.cuni.cz

**Keywords:** myokine, testosterone, cortisol, creatine kinase, bench press, interleukin, CRP, TNF-α

## Abstract

This study aimed to investigate the effects of a single bench press (BP) vs. leg press (LP) resistance training sessions on testosterone, cortisol, C-reactive protein (CRP) interleukin-6 (IL-6), tumor necrosis factor-α (TNF-α) concentrations, and creatine kinase (CK) activity in strength-trained males. Eleven strength-trained males participated in a cross-over randomized trial, undergoing two experimental sessions each consisting of five sets of the BP or the LP exercise to volitional failure with a load corresponding to 50% of one-repetition maximum. Blood samples were taken at baseline (BA), immediately post (POST), and 1 h after the cessation of exercise (POST-1). A significant increase in IL-6 concentration from BA to POST-1 was observed during the LP condition (*p* = 0.004; effect size [ES] = 0.64). Additionally, a significant main effect of time was found for increasing testosterone concentrations from BA to POST exercise (*p* = 0.014; ES = 0.25). A significantly lower cortisol concentration at POST-1 compared to POST (*p* = 0.001; ES = 1.02) was noted in the BP condition. Furthermore, a significantly lower cortisol concentration was found at POST-1 in the BP compared to the LP condition (*p* = 0.022; ES = 1.3). A significant increase in CK activity was reported from BA to POST (*p* = 0.024; ES = 0.69) and POST-1 (*p* = 0.045; ES = 0.55) during the LP condition, and from BA to POST-1 (*p* = 0.014; ES = 0.96) during the BP condition. No significant differences were found in the CRP (*p* = 0.659) and TNF-α concentrations (*p* = 0.487). These results suggest that the amount of muscle mass engaged during the resistance exercise may influence the changes in IL-6 and cortisol concentrations. Larger muscle groups, as engaged in the LP, more likely lead to elevated concentrations of IL-6 myokine.

## 1. Introduction

It is a well-known fact that resistance exercise can provide a potent stimulus for acute changes in endocrine responses and inflammatory reactions [1,2]. Resistance exercise is a stress factor that triggers a response from most systems of the human body, such as the cardiovascular, respiratory, immune, endocrine, and musculoskeletal systems [3]. Mechanisms of homeostatic control include the response of multiple regulators of the endocrine system such as the hypothalamic–corticotroph (pituitary) axis and locus caeruleus/norepinephrine system, hormones such as testosterone, prolactin, growth hormone, and the specific response of the cytokine system [4,5]. The physiological stress caused by intensive resistance exercise acts as a major stimulus for muscle fiber hypertrophy, and efficient muscle repair requires a well-coordinated and controlled inflammatory response [6]. A single bout of resistance exercise often increases circulating anabolic and catabolic hormones, as well as inflammatory cytokines [1,3]; however, some studies report no changes in these variables [7]. This variability in physiological responses is influenced by several factors, such as exercise intensity (% of one-repetition maximum [RM]), volume (i.e., number of repetitions and sets, duration), rest interval length, and the muscle mass involved (exercise selection and sequence) in the exercise [1,2]. A recent study by Kotikangas et al. [8] indicated a higher acute elevation in testosterone and cortisol concentration after five sets of 10 repetitions at 10 RM compared to seven sets of three repetitions at three RM of the Smith machine back squats. This suggests that it is the volume rather than the load of the resistance exercise that intensifies the acute increase in these hormones. In case of cytokines, Nieman et al. [9] reported a higher interleukin-6 (IL-6) concentration following running compared to cycling at similar intensities. On the one hand, this may be due to the greater amount of muscle mass that is recruited with running, meaning that more muscle fibers will be stimulated to produce IL-6. Alternatively, the greater IL-6 concentrations post-running may be a result of eccentric muscle contraction, resulting in increased muscle damage and IL-6 leaking into the circulation. Therefore, it seems that the highest acute increases in serum testosterone and cortisol, as well as inflammatory cytokines, have been reported following multi-set medium-intensity exercises, involving large muscle groups and performed close to volitional failure [1,2,10,11]. Such a protocol may impact the immune system in a similar manner to endurance exercise [10], but the exact mechanisms that induce these responses during resistance exercise are not well known [12,13].

There is considerably more data on the impact of exercise on the endocrine and inflammatory responses regarding aerobic exercise compared to resistance training [13,14,15]. The available research has primarily focused on comparing the impact of different resistance training intensities and volumes on changes in hormone [8,11] and cytokine concentrations [16], with little attention paid to the differences in the impact of upper- and lower-body exercises. Research suggests that the amount of muscle mass involved during exercise has an impact on the hormonal response [2]. For instance, exercises engaging large muscle mass have been shown to produce higher elevations in testosterone compared to small muscle mass exercises [2]. Nevertheless, there is lack of recent studies directly comparing changes in testosterone and cortisol concentrations following lower- vs. upper-body resistance training. For instance, Kraemer et al. [17] reported an increase in testosterone concentration after unilateral isometric knee extension but not after unilateral isometric bench press, both performed at 100% of maximum voluntary isometric contraction. However, some studies indicate that the type of muscle contraction influences post-exercise changes in hormone concentrations [18]; thus, it is not known whether the same results would be observed after isotonic exercise. Moreover, to the best of the authors’ knowledge, there is a lack of studies comparing changes in inflammatory responses following resistance exercises that differ in the involved muscle mass, while studies of inflammatory responses after different modes of exercise provide inconclusive data [3]. Therefore, it seems that the potential distinct magnitude of inflammation following lower- vs. upper-body exercises might depend on their duration.

To the best of the authors’ knowledge, there are no studies that have comprehensively and directly compared the acute hormonal and inflammatory responses following upper- and lower-body resistance exercise. Studying the extent of the acute hormonal and inflammatory response to various resistance exercises is crucial, as it might influence training adaptations. Furthermore, understanding the impact of upper and lower body exercises on cytokine responses is valuable in determining the significance of muscle mass, as cytokines are generated in the skeletal muscle [19]. Therefore, the degree of interaction between the endocrine and cytokine responses in trained individuals remains to be clarified. Consequently, this study aimed to assess the effects of a single upper-body (bench press) vs. lower-body (leg press) resistance training session on testosterone, cortisol, IL-6, c-reactive protein (CRP), TNF-α concentration, and creatine kinase (CK) activity, in strength-trained males. It was hypothesized that both protocols would contribute to a significant increase in testosterone, cortisol, IL-6 concentrations, and CK activity but the magnitude of this effect would be greater after the leg press compared to the bench press training session, due to the greater muscle mass involved in the exercise.

## 2. Results

Table 1 shows descriptive data for bench press and leg press conditions.

### 2.1. Inflammatory Markers

A related sample Friedman’s test by ranks did not show any significant differences in the CRP (test = 3.267; *p* = 0.659; W = 0.059) and TNF- α concentrations (test = 4.450; *p* = 0.487; W = 0.081). However, significant differences were found in the IL-6 concentration (test = 20.521; *p* = 0.001; W = 0.373). Pairwise comparison showed a significant increase from baseline to POST-1 in the IL-6 concentration following the leg press exercise protocol (*p* = 0.004; Δ = +0.92 pg/mL; ES = 0.64) (Table 2).

### 2.2. Hormonal Markers

**Figure 1:**
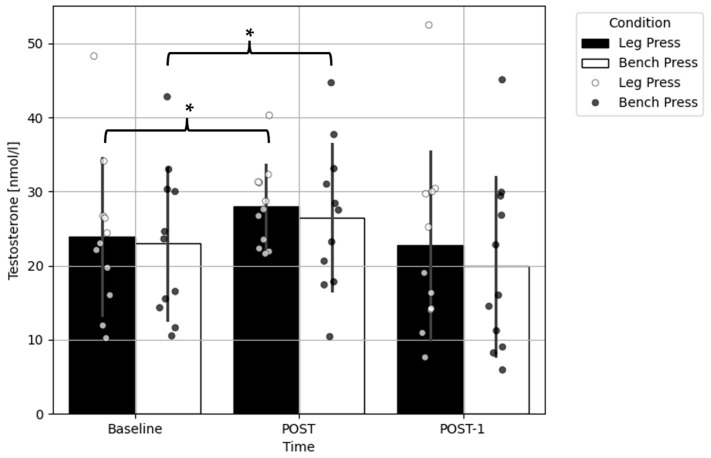
Comparison of the testosterone concentration before and after the bench press and leg press exercise protocols. Data presented as the mean ± standard deviation; * a significant difference compared to the baseline within the condition (*p* < 0.05); POST—immediately after completion of the exercises; POST-1—an hour after completion of the exercises.

The two-way ANOVA did not show a significant interaction (F = 0.669, *p* = 0.523) nor the main effect of condition (F = 0.960; *p* = 0.350), but it did show a significant main effect of time (F = 11.030; *p* = 0.001) for the testosterone concentration. A pairwise comparison revealed a significant increase in testosterone concentrations from baseline to POST exercise (*p* = 0.014; Δ = +3.8 nmol/L; ES = 0.25) (Figure 1).

**Figure 2:**
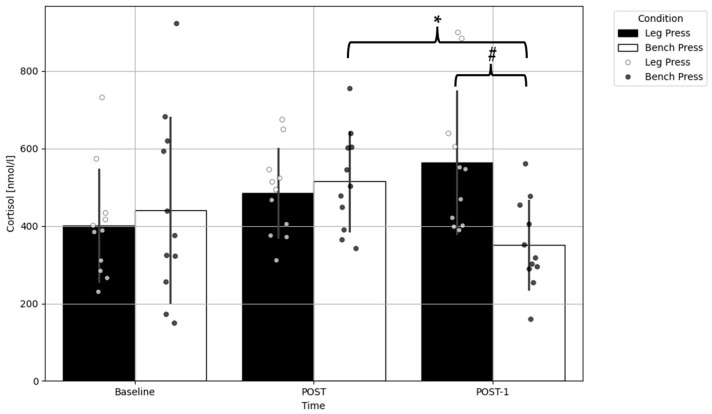
Comparison of the cortisol concentration before and after the bench press and leg press exercise protocols. Data presented as mean ± standard deviation; * a significant difference compared to the POST within condition (*p* < 0.01); # a significant difference compared to leg press within time point (*p* < 0.05); POST—immediately after completion of the exercises; POST-1—an hour after completion of the exercises.

Moreover, a significant interaction was found for the cortisol concentration (F = 8.467, *p* = 0.002). Pairwise comparisons showed a significantly lower cortisol concentration at POST-1 compared to POST (*p* = 0.001; Δ = −74 nmol/L; ES = 1.02) in the bench press condition. Furthermore, a significantly lower cortisol concentration was found at POST-1 in the bench press exercise compared to the leg press condition (*p* = 0.022; Δ = −213 nmol/L; ES = 1.3) (Figure 2).

### 2.3. Creatine Kinase Activity

A related sample Friedman’s test by ranks showed significant differences in the CK activity (test = 27.995; *p* < 0.001; W = 0.51). Pairwise comparison showed a significant increase in CK activity from baseline to POST (*p* = 0.024; Δ = +87 U/L; ES = 0.69) and POST-1 (*p* = 0.045; Δ = +72 U/L; ES = 0.55) during the leg press condition, and from BA to POST-1 (*p* = 0.014; Δ = +125 U/L; ES = 0.96) during the bench press condition (Table 3).

## 3. Discussion

This study aimed to evaluate the acute effects of leg press and bench press single training session on the CRP, IL-6, TNF-α, CK activity, testosterone, and cortisol concentration changes. The main finding revealed an acute increase in testosterone concentration immediately after both conditions. However, at the POST-1 measurement point, there was no significant difference compared to baseline values in either condition. The only distinct responses following the leg press and bench press exercise protocols were pronounced in IL-6 and cortisol concentration. The Il-6 concentration significantly increased only following the leg press exercise at POST-1, whereas cortisol concentrations significantly decreased in POST-1 compared to POST after the bench press exercise protocol. Additionally, the CK activity increased significantly from baseline following both conditions. In the bench press condition, the increase was significant only at POST-1, while for the leg press exercise, a significant increase was observed at both POST and POST-1. The CRP and TNF-α concentrations remained unchanged in both conditions and time points.

The obtained results only partially confirmed our hypothesis of a significant increase in the studied variables with a greater magnitude effect following the leg press compared to the bench press training session. It was demonstrated that immediate post-training responses after upper- and lower-body training significantly differed only in the case of IL-6 and cortisol concentrations. A significant increase in IL-6 concentration was noted after the lower-body condition at POST-1, but this finding was not observed in the bench press exercise. Another distinct reaction was a significant decrease in cortisol concentrations after the bench press exercise protocol. In contrast, following the leg press exercise, the cortisol concentration gradually increased but did not reach statistical significance. These results suggest that the amount of muscle mass engaged during exercise may differentiate the extent of changes in IL-6 and cortisol concentrations [20]. Glycogen depletion and tissue damage in exercising skeletal muscle may stimulate IL-6 release. Considering the significant and comparable increase in CK activity after both leg press and bench press conditions, it seems that the observed increase in IL-6 concentration following the leg press condition was less related to muscle damage but perhaps more to muscle glycogen depletion [21]. Previous studies have shown that several sets of resistance exercise significantly reduce glycogen levels [22,23] and the largest reductions are observed following an exercise protocol with a high number of repetitions and moderate loading (50–60%1 RM) [24]. The leg press exercise generally involves a larger muscle mass compared to the bench press. Leg press primarily targets the muscles in the lower body, including quadriceps, hamstrings, glutes, and calves. On the other hand, the bench press primarily targets the muscles in the upper body, particularly the chest, shoulders, and triceps. Larger muscle groups, as engaged in leg press, are likely to experience more considerable glycogen depletion leading to elevated concentrations of markers such as IL-6. In contrast, a study by Lira et al. [25] found no differences in IL-6, cortisol, and TNF-α concentrations after four bouts of lower and upper Wingate tests among judo athletes. The different results compared to Lira et al. [25] might be due to the type of exercises used in the study protocols. While both protocols involved all-out efforts, during cycling, the eccentric component is limited, whereas eccentric contractions cause greater exercise-induced muscle damage than other types of muscle contractions. This could also explain a higher IL-6 concentration following running compared to cycling at similar intensities in the study by Nieman et al. [9]. Unfortunately, both Lira et al. [25] and Nieman et al. [9] did not assess CK activity.

In addition to the increase in IL-6 concentrations after the leg press condition, no significant changes in the CRP and TNF-α concentrations were observed after both conditions. In general, TNF-α is classified as a leading pro-inflammatory cytokine, but unlike in sepsis, its concentrations increase marginally in the acute response after exercise [26]. Additionally, it has been demonstrated that IL-6 is expressed in contracting muscle fibers [27] and released from skeletal muscle during exercise, whereas this is not the case for TNF-α. Moreover, exercise-induced increases in IL-6 exert inhibitory effects on TNF-α production. Therefore, the lack of changes in TNF-α concentration in this study seems reasonable. On the other hand, the synthesis of CRP is stimulated by IL-6 [28], although no changes were observed in this study either at POST as well as at POST-1. However, considering the elevated IL-6 and CK concentrations, it is likely that the CRP concentration increased but at a later time (>8 h) [29]. For example, such a phenomenon was observed in the study by Mendham et al. [30]. The authors reported a significant increase in IL-6 and no changes in CRP concentration immediately after a whole-body resistance training session (7 exercises performed in 3 sets of 10 repetitions at 80% 1 RM). However, 24 h after the training session, the CRP concentration significantly increased, while IL-6 returned to baseline levels. Such results may suggest that IL-6 is an earlier and more prominent inflammatory marker after physical exercise than CRP within an immediate exercise bout.

In this study, a significant increase in testosterone concentration was found, which returned to baseline (even slightly below) at POST-1. Changes in cortisol concentrations at POST-1 differed between conditions. In both exercise protocols, the cortisol concentration showed an upward trend at POST but did not reach statistical significance. After the bench press exercise, cortisol significantly decreased to concentrations below the POST value, while after the leg press condition, it maintained a non-significant upward trend. In contrast, Geisler et al. [31] showed a significant increase in testosterone concentration up to 45 min after completing barbell back squats (5 sets of 10 repetitions at 75% 1 RM), as well as in cortisol 15 and 45 min post-exercise. Following the same bench press protocol, no significant changes in the testosterone and cortisol levels were observed. Similarly, Mendham et al. [30] noted an increase immediately post-exercise, followed by a return to baseline. In contrast, Izquierdo et al. [1] reported an acute testosterone concentration increase following 5 sets of 10 RM (~75% 1 RM) leg press immediately post-exercise, returning to baseline 45 min later. Cortisol concentrations did not change immediately post-exercise but significantly increased at 15 and 45 min compared to baseline. Studies on the acute effects of resistance exercise on testosterone and cortisol concentrations are inconsistent. One explanation may be due to the participants’ training background which may cause differences in the magnitude of acute responses in endocrine systems following strenuous exercises [8]. Participants in the mentioned studies differed significantly in the level of muscular strength. In Izquierdo et al. [1], participants’ 1 RM was approximately 190.6 ± 30.2 kg in the leg press exercise, while in our study, the participants’ 1 RM was considerably higher, namely 327 ± 36 kg. In Mendham et al. [30], 1 RM results were not provided, but participants were characterized as sedentary, and hence probably less trained. An alternative explanation for the different results might be the significantly lower training intensity and higher volume used in this study compared to those mentioned above (50% 1 RM vs. ~75% 1 RM); and 5 sets until volitional failure, resulting in slightly above 100 repetitions vs. 5 sets of 10 repetitions in the studies by [1,31]. In contrast, in the study by Mendham et al. [30], where the responses regarding IL-6, CRP, and cortisol concentrations were similar to those in this study, the volume was the highest (7 exercises performed in 3 sets of 10 repetitions at 80% 1 RM). This has been also confirmed by studies that directly compared the effects of different resistance training volumes and intensities on testosterone and cortisol levels. A recent study by Kotikangas et al. [8] indicated a higher acute elevation in testosterone and cortisol after 5 sets of 10 repetitions at 10 RM compared to 7 sets of 3 repetitions at 3 RM of Smith machine back squats. Hakkinen and Pakarinen [11] reported a significantly higher testosterone and cortisol increase after 10 sets of 10 repetitions at 70% 1 RM than after 20 sets of a single repetition at 1 RM of back squats. This suggests that it is the volume rather than the intensity of the resistance exercise that enhances the acute secretion of these hormones.

There are a number of limitations to this study. Firstly, only a single exercise, volume, and intensity were assessed, and only in male participants. Additionally, blood samples were taken immediately after and one-hour post-exercise, limiting the ability to extrapolate findings to other exercises, volumes, intensities, groups, and later responses. Furthermore, it should be noted that the comparison in this study was made between machine (leg press) vs. free weight (bench press) exercises, which might have impacted the findings. For instance, Shaner et al. [32] reported that training sessions with free-weight squat exercises result in greater acute release of anabolic hormones, such as testosterone and growth hormone, compared with the more stable leg press exercise. Additionally, free weight exercises, such as the back squats with a significant eccentric component, cause a much greater muscle damage and inflammation compared with exercises performed on machines. Another limitation is that the study was conducted on small sample size. Ultimately, small sample sizes can hinder the ability to detect true effects and draw reliable conclusions. It is important to be aware of these limitations when interpreting and generalizing study findings Therefore, future research should focus on evaluating the impact of other exercises on immediate and long-term physiological responses, considering a broader panel of biomarkers (e.g., hormonal, markers of damage and inflammation). Additionally, these studies should include other groups of participants, such as women and mixed-sex groups.

## 4. Materials and Methods

### 4.1. Experimental Approach to the Problem

This study was performed following a randomized, crossover design, where each participant underwent two experimental sessions to compare the acute responses of the bench press and leg press exercises performed to volitional failure on testosterone, cortisol, IL-6, CRP, TNF-α concentration, and CK activity. During each training session the participants performed five sets to volitional failure at 50% 1 RM with a 3 min rest interval between sets. Blood samples were taken at baseline, immediately post and 1 h after the last set.

### 4.2. Participants

Eleven resistance-trained males (age: 23 ± 2 years; body height: 180 ± 9 cm; body mass: 81.4 ± 8.6 kg; body fat percentage: 14.7 ± 5.9%; training experience: 6 ± 2 years; leg press 1 RM: 327 ± 36 kg; bench press 1 RM: 95 ± 14 kg) participated in this study. The inclusion criteria for both groups were as follows: (i) no lower-limb serious injury, including tendon or muscle tear; and (ii) participation in regular resistance training in the previous 2 years. The study consisted of 3 sessions: a familiarization session and two experimental sessions one week apart. Participants were advised to adhere to their regular sleep hygiene and dietary habits and to avoid stimulants or any ergogenic aids throughout the study. All sessions were consistently scheduled at the same time of the day (9:00–11:00) to mitigate the influence of circadian rhythm. Additionally, participants were requested to abstain from additional high-intensity exercise 72 h prior to testing to minimize fatigue. The participants were informed about the study’s benefits and potential risks before the experiment’s commencement and gave their written consent to participate. The study protocol was approved by the Bioethics Committee for Scientific Research at the Academy of Physical Education in Katowice, Poland (2/2014), and performed according to the ethical standards of the Declaration of Helsinki 2013.

### 4.3. Familiarization Session

During the familiarization session, the participants underwent 1 RM tests for the bench press and leg press exercises in a randomized order. The session began with a warm-up, including 5 min of cycling at approximately 70 rpm with a load of 1 W per kg of body mass, followed by dynamic exercises: 2 sets of 5 repetitions each for bodyweight squats, lunges (on each lower limb), push-ups, and resistance band standing rows (on each upper limb). Subsequently, the 1 RM test for either the bench press or leg press commenced. The participants completed 15, 10, and 5 repetitions at 20%, 40%, and 60% of their estimated 1 RM, respectively. The initial testing load was set at an estimated 80% of 1 RM, and subsequent attempts for the bench press increased by 2.5–5 kg, while for the leg press, they increased by 10–20 kg until reaching volitional failure. A 3 min rest interval between successful sets was allowed. For the bench press, hand placement on the barbell was set at 150% of the individual’s bi-acromial distance, and the hand positioning was recorded to maintain consistency across experimental sessions. The leg press exercise was conducted using the Keiser Leg Press A420 pneumatic device (Keiser, Fresno, CA, USA). The test was performed from a seated position with approximately 90° knee flexion and feet flat on each footplate. For both exercises, participants were instructed to complete each repetition with maximum velocity, emphasizing a “push as fast as possible” approach.

### 4.4. Experimental Sessions

After the warm-up (same as in the familiarization session) and before the main exercises, blood samples were collected immediately following, as well as an hour after, the completion of the exercise protocols for the evaluation of selected blood variables. At each time point, venous blood samples were collected into 4 mL tubes. Blood was drawn in a sitting position. The main exercise protocol consisted of 5 sets of the bench press or the leg press exercise performed to volitional failure with a load corresponding to 50% of 1 RM and a 3 min rest interval between sets. The reason for selecting this intensity was that previous research had shown that a high training volume at medium intensities caused more physiological disturbances than high intensities [8,11]. Additionally, this intensity closely aligns with the optimal load for maximizing power output [33] and also appears to be safe for participants, particularly when sets are performed until volitional failure. The number of performed repetitions and peak power obtained in each set were registered. The peak power value was obtained as the mean of the peak power output obtained in each repetition of a given set.

### 4.5. Biochemical Analysis

Blood was placed in the test tubes to separate plasma (BD Vacutainer PPT™ Plasma Preparation Tube, Becton, Dickinson and Company, Plymouth, UK) and serum (BD Vacutainer™ Serum Tube, Becton, Dickinson and Company, Plymouth, UK). Plasma was obtained by centrifuging the tubes for 10 min at 1000× *g* at 4 °C (SIGMA 2-16KL, Sigma Laborzentrifugen GmbH, Osterode am Harz, Germany). To extract serum, the test tubes were allowed to stand for 30 min for blood to clot and then were centrifuged at 1000× *g* at 4° C. Serum was stored at −80 °C for less than one month before being assayed.

The CK activity (CK, 2.7.3.2) in fresh plasma samples was determined using the Randox Laboratories diagnostic kit (no. CK522, Randox Laboratories, Crumlin, UK). The intra- and inter-assay coefficients of variation (CV) were 1.93% and 3.63%. The IL-6 and TNF-α concentrations were assayed using sandwich enzyme immunoassay kits (no. 950.035.096 for IL-6 and 950.090.96 for TNF-α, Diaclone SAS, Besançon, France). The intra- and inter-assay CVs for IL-6 were 4.4% and 9.1% and for TNF-α concentrations, these were 3.2% and 10.9%, respectively.

The testosterone and cortisol concentrations were assayed by radioimmunoassay kits (no. RK-61 CT and no. RK-240 CT, Izotop, Institute of isotopes Co., Ltd., Budapest, Hungary). The intra- and inter-assay CVs were 5.8% and 8.9% for testosterone, and 3.1% and 4.6% for cortisol concentrations, respectively.

The concentrations of CRP were determined using the ADIVIA 1800 clinical chemistry system (Siemens Healthineers, Erlangen, Germany).

### 4.6. Statistical Analysis

All statistical analyses were performed using SPSS (version 25.0; SPSS, Inc., Chicago, IL, USA) and were shown to be a means with standard deviations (±SD). Statistical significance was set at *p* < 0.05. The normality of data distribution was checked using Shapiro–Wilk tests, and Mauchly’s test was used to test for the assumption of sphericity. The two-way ANOVAs with repeated measures (2 conditions [bench press, leg press] × 3 time points [baseline, POST, POST-1]) were used to investigate the effects of each training session on selected blood variables. When a significant main effect or interaction was found, the post hoc tests with Bonferroni correction were used to analyze the pairwise comparisons. If data normality distribution was not confirmed, related-samples Friedman’s test by ranks were used, and the effect size was estimated by Kendall’s coefficient of concordance. The magnitude of mean differences was expressed with standardized effect sizes. Thresholds for the qualitative descriptors of Hedges g were interpreted as ≤0.20 “small”, 0.21–0.79 “medium”, and >0.80 as “large” [34].

## 5. Conclusions

The results of this study indicate that both studied exercise protocols, i.e., five sets to volitional failure of leg press and bench press at 50% 1 RM, led to an acute, short-term increase in testosterone concentration, which returned to baseline levels after one hour. The CK activity significantly increased, while the CRP and TNF-α concentrations remained unchanged in both conditions. Significant differences in the studied blood markers between conditions were observed only in the IL-6 and cortisol concentrations. The Il-6 concentration significantly increased only in the leg press condition at POST-1, whereas the cortisol concentrations significantly decreased at POST-1 compared to POST after the bench press exercise. These results suggest that the amount of muscle mass engaged during exercise may influence changes in the IL-6 and cortisol concentrations. Larger muscle groups, as engaged in the leg press exercise, most likely lead to elevated concentrations of markers such as IL-6 myokine.

## Figures and Tables

**Table 1:** Descriptive data for the bench press and leg press exercise performance.

Condition	Set 1 (95%CI)	Set 2 (95%CI)	Set 3 (95%CI)	Set 4 (95%CI)	Set 5 (95%CI)
Number of Repetitions [n]					
Bench press	37 ± 5 (33 to 41)	23 ± 5 (20 to 26)	19 ± 4 (16 to 21)	16 ± 5 (13 to 19)	14 ± 4 (12 to 17)
Leg press	34 ± 4 (31 to 36)	25 ± 6 (21 to 30)	21 ± 7 (16 to 26)	20 ± 6 (16 to 24)	20 ± 4 (17 to 22)
Peak Power [W]					
Bench press	628 ± 186 (503 to 753)	570 ± 128 (484 to 656)	546 ± 126 (461 to 631)	492 ± 143 (396 to 589)	480 ± 127 (395 to 565)
Leg press	1134 ± 160 (1027 to 1242)	1010 ± 160 (902 to 1117)	891 ± 153 (788 to 993)	834 ± 143 (738 to 930)	795 ± 167 (683 to 907)

Data presented as mean ± standard deviation. CI—confidence intervals.

**Table 2:** Comparison of inflammatory markers before and after the bench press and leg press exercise protocols.

	Baseline (95%CI) [Mdn]	POST (95%CI) [Mdn]	POST-1 (95%CI) [Mdn]
CRP [mg/L]			
Bench press	3.9 ± 1.2 (3.1–4.7) [3.8]	3.8 ± 1.6 (2.8–4.9) [3.5]	4.0 ± 2.2 (2.5–5.5) [3.8]
Leg press	3.6 ± 1.7 (2.5–4.7) [3.4]	3.8 ± 1.9 (2.5–5.0) [3.3]	3.5 ± 1.9 (2.2–4.7) [2.9]
IL-6 [pg/mL]			
Bench press	1.18 ± 0.96 (0.54–1.82) [0.7]	1.35 ± 1.19 (0.55–2.15) [0.85]	1.29 ± 0.62 (0.87–1.71) [1.17]
Leg press	1.58 ± 1.77 (0.39 to 2.77) [0.55]	1.92 ± 1.75 (0.75–3.09) [0.97]	2.49 ± 1.86 * (1.25–3.74) [1.79]
TNF-α [pg/mL]			
Bench Press	23.36 ± 3.78 (20.81–25.90) [21.90]	23.43 ± 4.46 (20.43– 26.42) [21.90]	22.63 ± 3.16 (20.51–24.75) [21.46]
Leg Press	22.67 ± 4.34 (19.75–22.58) [20.81]	22.77 ± 4.10 (20.01–25.52) [20.81]	22.72 ± 4.02 (22.02–25.43) [21.25]

Data presented as mean ± standard deviation. CI—confidence interval; Mdn—median; * a significant difference compared to baseline within condition (*p* < 0.01); POST—immediately after completion of the exercises; POST-1—an hour after completion of the exercises.

**Table 3:** Comparison of CK activity before and after the bench press and leg press exercise protocols.

	Baseline (95%CI) [Mdn]	POST (95%CI) [Mdn]	POST-1 (95%CI) [Mdn]
Creatine Kinase [U/L] (95%CI [Mdn])			
Bench press	159 ± 63 (117–201) [154]	237 ± 162 (128–346) [205]	284 ± 202 * (149–420) [219]
Leg press	170 ± 80 (116–225) [154]	262 ± 118 * (183–341) [237]	242 ± 107 * (170–314) [210]

Data presented as mean ± standard deviation. CI—confidence interval; Mdn—median; * a significant difference compared to baseline within condition (*p* < 0.05).

## Data Availability

The datasets analyzed during the current study are available from the corresponding author upon reasonable request.
